# MC38 colorectal tumor cell lines from two different sources display substantial differences in transcriptome, mutanome and neoantigen expression

**DOI:** 10.3389/fimmu.2023.1102282

**Published:** 2023-03-08

**Authors:** Barbara Schrörs, Brett J. Hos, Ikra G. Yildiz, Martin Löwer, Franziska Lang, Christoph Holtsträter, Julia Becker, Mathias Vormehr, Ugur Sahin, Ferry Ossendorp, Mustafa Diken

**Affiliations:** ^1^ TRON - Translational Oncology at the University Medical Center of the Johannes Gutenberg-University Mainz gGmbH, Mainz, Germany; ^2^ Department of Immunology, Leiden University Medical Center, Leiden, Netherlands; ^3^ BioNTech SE, Mainz, Germany; ^4^ Research Center for Immunotherapy (FZI), University Medical Center of the Johannes Gutenberg University Mainz, Mainz, Germany

**Keywords:** MC38, colorectal carcinoma, mutation analysis, neoantigens, expression profile, murine tumor model

## Abstract

**Introduction:**

The cell line MC38 is a commonly used murine model for colorectal carcinoma. It has a high mutational burden, is sensitive to immune checkpoint immunotherapy and endogenous CD8+ T cell responses against neoantigens have been reported.

**Methods:**

Here, we re-sequenced exomes and transcriptomes of MC38 cells from two different sources, namely Kerafast (originating from NCI/NIH, MC38-K) and the Leiden University Medical Center cell line collection (MC38-L), comparing the cell lines on the genomic and transcriptomic level and analyzing their recognition by CD8+ T cells with known neo-epitope specificity.

**Results:**

The data reveals a distinct structural composition of MC38-K and MC38-L cell line genomes and different ploidies. Further, the MC38-L cell line harbored about 1.3-fold more single nucleotide variations and small insertions and deletions than the MC38-K cell line. In addition, the observed mutational signatures differed; only 35.3% of the non-synonymous variants and 5.4% of the fusion gene events were shared. Transcript expression values of both cell lines correlated strongly (p = 0.919), but we found different pathways enriched in the genes that were differentially upregulated in the MC38-L or MC38-K cells, respectively. Our data show that previously described neoantigens in the MC38 model such as Rpl18^mut^ and Adpgk^mut^ were absent in the MC38-K cell line resulting that such neoantigen-specific CD8+ T cells recognizing and killing MC38-L cells did not recognize or kill MC38-K cells.

**Conclusion:**

This strongly indicates that at least two sub-cell lines of MC38 exist in the field and underlines the importance of meticulous tracking of investigated cell lines to obtain reproducible results, and for correct interpretation of the immunological data without artifacts. We present our analyses as a reference for researchers to select the appropriate sub-cell line for their own studies.

## 1 Introduction

Effective immunotherapy with immune checkpoint inhibitors (ICIs) correlates with the mutational burden of treated tumors (1–4). High rates of tumor-specific mutations improve the odds of MHC class I-presented mutated peptide sequences, which, due to the lack of immunologic tolerance to such neoantigens, are more likely to be recognized by T cells as non-self. Specific T cell responses have been identified against neoantigens in cancer patients, and ICIs are effective in the stimulation of neoantigen-specific responses (5–10). The relevance of this class of cancer antigens is also supported by observations that tumors are under constant immunological pressure against neoantigens, and ICIs induce a marked shift of expressed neoepitopes (9, 11–13).

The identification of immunologically relevant neoantigens has become a feasible exercise due to recent technological advancements in whole-genome and -exome sequencing. These technologies are suitable for the identification of expressed non-synonymous variations (SNVs), frameshift mutations, and fusion genes. We and others, have successfully used this approach to identify mutation-derived epitopes in (pre-)clinical settings for the design of neoantigen-specific cancer vaccines (14–20).

The MC38 adenocarcinoma colorectal cell line is a well-established and often used tumor model for pre-clinical studies of neoantigens and immunotherapeutic approaches (13, 21–26). This transplantable cell line was established in 1975 by repeated injection of the carcinogen di-methyl hydrazine in mice and is therefore characteristic of a tumor with high mutational burden (27). Recently, this cell line was sequenced for the identification of several immunogenic neoepitopes by Yadav and colleagues (14). Our own research identified an additional mutation in the Rpl18 gene that instigated a dominant endogenous CD8+ T cell response, while the previously identified epitope in the Adpgk gene appeared less dominant (20). Most of the mutations described by Yadav et al. (14) we could confirm, which was obviously the result of the same (Leiden) origin of the MC38 cell line in both studies. This MC38-L cell line was in the possession of the Leiden laboratory since the mid-1990s. However, another publicly available MC38 cell line from Kerafast (NCI/NIH origin, MC38-K) appeared to lack expression of the published immunogenic mutations, as this cell line failed to activate our MC38-L-specific T cell lines in coculture. This raised questions about the genetic constitution and altered immunogenicity of this MC38 cell line, since the MC38-K cell line is also commonly used for immunotherapeutic studies (26, 28).

In this study, we re-sequenced the MC38-L and MC38-K cell lines for whole-exome and transcriptomic comparison. We found major discrepancies in the mutational landscape and distinct pathways were upregulated in the MC38-L or MC38-K cells which might be relevant for proposed onco-immunological studies. Several previously identified immunogenic neoantigens (i.e. mutated Rpl18 and mutated Adpgk) were lacking in the MC38-K cell line, thus only the MC38-L cell line was recognized by these neoantigen-specific T cells. These findings underscore the importance of the accurate sourcing of tumor cell lines which are commonly used in the immunotherapeutic field.

## 2 Methods

### 2.1 Samples

#### 2.1.1 Animals

Female C57BL/6 Thy1.1^+^ donor mice were purchased from Envigo. All mice were kept in accordance with federal and state policies on animal research at BioNTech SE, Germany.

#### 2.1.2 Cell lines, culture conditions and generation of viral supernatant

MC38-L and MC38-K colon carcinoma cell lines were provided by Leiden University Medical Center, Netherlands, and Kerafast, USA, respectively, and cultured under standard conditions. MC38-L cells were cultured in IMDM (ATCC, 30-2005) containing 8% Fetal Bovine Serum (FBS), 2 mM L-glutamine and 50 µM beta-mercaptoethanol. MC38-K cells were cultured in DMEM (ATCC, 30-2002) supplemented with 10% FBS, 10 mM HEPES and 1X nonessential amino acids (NEAA). B16-Ova melanoma cell line, ectopically expressing ovalbumin antigen, was a gift from Udo Hartwig (University Medical Center Mainz, Germany) and cultured in DMEM (Gibco) containing 10% FBS. Platinum-E cells were used for generation of MLV-E pseudotyped viral particles for different TCRs and maintained under standard conditions in DMEM (Gibco) supplemented with 10% FBS. The cells were transfected with TransIT-LT1 (Mirus) based on manufacturer´s instructions. Retroviral supernatants were collected 48 and 72 h after transfection. The titers were determined using mCAT cells as described in (29).

### 2.2 Bioinformatics analyses

#### 2.2.1 High-throughput sequencing and read alignment

Exome capture from MC38 cell lines and C57BL/6 mice were sequenced in duplicate using the Agilent Sure Select Kit and Agilent SureSelectXT Mouse All Exon exome capture assay. Oligo(dT)-isolated RNA for gene expression profiling of the MC38 cell lines was prepared in duplicate with Illumina’s TruSeq stranded Library Prep Kit. Libraries were sequenced on an Illumina HiSeq2500 or NovaSeq6000 (2x50 nt). DNA-derived sequence reads were aligned to the mm9 genome using bwa [(30); default options, 0.7.10]. RNA-derived sequence reads were aligned to the mm9 genome using STAR [(31); default options, version 2.1.4a]. The sequencing reads are available in the European Nucleotide Archive (see Data Availability Statement).

#### 2.2.2 Mutation detection

Strelka2 [(32); default options for whole exome sequencing, version 2.9.9] was used to call somatic SNV and short insertion/deletion (indel) on each cell line or normal library replicate pair individually.

#### 2.2.3 DNA copy number calling

Absolute copy numbers were called from exome capture data as described before (33) using Control-FREEC [(34); version 11.5].

#### 2.2.4 Mutation signatures

Mutation signatures (35) were computed with the R package YAPSA [(36); default settings, version 1.10.0].

#### 2.2.5 Fusion gene detection

Fusion genes were detected with EasyFuse (version 1.3.6) using a “wisdom of crowds” approach as detailed before (37). Entries in the “references” and “other_files” sections of the EasyFuse configuration were changed to Ensembl GRCm38.95. Data for both MC38 cell lines was available in two replicates. Intersection of fusion gene events [i.e. unique breakpoint IDs (BPID)] from both replicates with a prediction probability score ≥ 0.5 was taken from each origin to obtain a high confidence dataset. Fusion events reported in chrY were not considered.

#### 2.2.6 Circos plots

Somatic alterations in each cell line (SNVs, INDELs, fusion genes and copy number variations) were visualized in circos plots with R package Circlize [(38); version 0.4.11]. Genomic coordinates of the fusion event breakpoints were converted to mm9 with liftOver (39). Breakpoint 1 of the fusion event with BPID “X:170018795:+_X:169984999:+” could not be converted. For the visualization, it was manually set to X:166456727 at the same genomic distance to breakpoint 2 (X:166422931) in mm9.

#### 2.2.7 Transcriptome profiling

Transcript abundance estimation was done with kallisto [(40); default options, version 0.42.4] on each cell line library replicate individually using the mean transcripts per million (TPM) per transcript final value. Differential expression analysis was performed using DESeq2 [(41); version 1.24.0] with MC38-L cell line as “control” and the transcript counts reported by kallisto, summarized by adding up the counts of the respective transcripts associated with each gene. Enriched pathways (KEGG 2019 Mouse) in differentially up- or downregulated genes were determined using Enrichr (42).

### 2.3 Engineering of antigen specific murine T cells and immunogenicity testing

#### 2.3.1 Construction of T cell receptor vectors

The codon-optimized and synthesized individual TCR-alpha and TCR-beta sequences reactive against Adpgk_R304M_, Rpl18_Q125R_ and Ova_257-264_ antigens (Eurofins Genomics) were cloned into the retroviral vector MP71 for stable expression in murine T cells. TCR genes were connected to firefly luciferase and eGFP reporter genes by 2A-splice elements (43).

#### 2.3.2 Retroviral engineering of murine T cells

Splenocytes of naïve C57BL/6-Thy1.1+ mice were pre-activated by 2 mM/mL Concanavalin A (ConA) (Sigma) in T cell media, RPMI 1640-GlutaMAX supplemented with 10% FBS, 1x NEAA, 1 mM sodium pyruvate, 10 mM HEPES, 50 μM β-Mercaptoethanol, 50 IU/mL Penicillin and 50 μg/mL Streptomycin (all Gibco), in the presence of 450 IU/mL rh IL-7 and 50 IU/mL rh IL-15 (both Miltenyi). 24 h after activation, cells were gently spun down (1h, 37°C, 300 x *g*) and incubated on MLV-E-pseudotyped gamma-retroviral vector pre-coated-RetroNectin-plates (Takara). After additional overnight cultivation, spin-down transduction was repeated on freshly coated plates with viral particles. 72 h after initial pre-activation, ConA was removed from culture and lymphocyte layer was isolated by Ficoll-Hypaque (Amersham Biosciences) density gradient centrifugation. Non-transduced T cells used as control for some experiments underwent the same ConA-activation procedure. Transgene expression on transduced murine T cells were measured *via* flow cytometry.

#### 2.3.3 RNA constructs and *in vitro* transcription

Plasmid templates for *in vitro* transcription of antigen-encoding RNAs, i.e. Adpgk-RNA and Rpl18-RNA, were based on pSTI vector. They were designed to encode 27 amino acids with the mutated amino acid at the central position (position 14). As a control, OvaI-RNA encoding for Ova_257-264_ (SIINFEKL) peptide as enhanced green fluorescent protein (eGFP) was employed (44). *In vitro* transcription and capping with β-S-anti-reverse cap analog (ARCA) was performed as described in (45).

#### 2.3.4 Electroporation of target cells

MC38-L and MC38-K cells were resuspended in X-VIVO 15 (Lonza) and electroporated in 4-mm cuvettes (Bio-Rad) with an ECM 830 Square Wave Electroporation System (BTX) (300V, 15 ms, 1 pulse) after addition of 2 µg antigen encoding RNA. The cells were co-electroporated with 2 µg eGFP RNA as an electroporation control. Cells were diluted immediately in culture medium directly after electroporation. 16-20 h post electroporation, cells were harvested to be used in the downstream applications such as IFNγ ELISPOT or cytotoxicity assay. The transfection efficiency was assessed based on GFP expression *via* flow cytometry.

#### 2.3.5 Flow cytometry

Transduction efficiency and TCR expression by T cells following transduction was measured *via* flow cytometry. The monoclonal antibodies against mouse CD8α-PE-Cy7 (BioLegend; clone:53-67), CD8α-PE-Cy7 (ThermoFisher; clone: 5H10), and CD8α-APC-R700 (BD Biosciences; clone: 53-67) were used. Cytokine production by T cell lines was analyzed with TNFα-MP6-XT22 (BioLegend; clone: MP6-XT22) and IFNγ-PE-Cy7 (BD Biosciences; clone XMG1.2) antibodies. TCR expression after transduction was evaluated based on tetramer staining. The following tetramers were used; Adpgk-tetramer-APC (ASMTNMELM-H-2-D^b^), Adpgk-tetramer-PE (ASMTNMELM-H-2-D^b^), Rpl18-tetramer-APC (KILTFDRL-H-2-K^b^) and OvaI-tetramer-APC (SIINFEKL-H-2-K^b^) (all MBL). TCR transduced T cells were stained for 30 min at 4°C. PBS containing 5% FBS and 5 mM EDTA was used as washing and staining buffer. Acquisition and analysis were performed on BD FACS CantoII and FlowJo softwares, respectively.

#### 2.3.6 MC38-L-specific T cells

T cell lines originate from anti-PDL1 (clone MIH-5) treated, MC38 immune mice as described by Sow et al. (46), and *ex vivo* established *via* coculture of splenocytes with irradiated MC38 in IL2 supplemented (5 Cetus Units) medium [as described by Hos et al., 2019 (20)]. Recognition of live MC38 cells (MC38-L and MC38-K) by T cell lines was determined by cytokine production after o/n coculture in a 5:1 (effector: target) ratio and 2 µg/mL brefeldin A (Sigma-Aldrich) by IFNγ ELISPOT.

Adpgk-, Rpl18- or OTI-TCR-transduced T cells were cultured overnight at 37°C on anti-IFNγ (Mabtech, clone: AN18) pre-coated Multiscreen filter plates (Merck Millipore). 1x10^5^ transduced T cells were stimulated with 5x10^4^ tumor cells, i.e. B16-Ova, MC38-L or –MC38-K cells (untreated or pre-treated overnight with 20 ng/mL IFNγ), or MC38-K electroporated with Adpgk-, Rpl18- or OvaI-RNA. The spots were visualized with a biotin-conjugated anti-IFNγ antibody (Mabtech) followed by incubation with ExtrAvidin-Alkaline Phosphatase (Sigma-Aldrich) and BCIP/NBT substrate (Sigma-Aldrich). Plates were scanned using CTL’s ImmunoSpot^®^ Series S five Versa ELISpot Analyzer (S5Versa-02-9038) and analyzed by ImmunoCapture V6.3 software. The samples were tested in duplicates and spot counts were summarized as means of technical duplicates.

#### 2.3.7 Cytotoxicity assay

TCR mediated cytotoxicity was evaluated using the xCELLigence system (OMNI Life Science). Cell index (CI) impedance measurements were performed according to manufacturer´s instructions. Target cells MC38-L and MC38-K were seeded at a concentration of 4x10^4^ and 2x10^4^ cells per well, respectively, in E-plate 96 (ACES Biosciences Inc.). After 20-24 h, TCR transduced murine T cells were added at 60:1 E:T (effector:target) ratio onto tumor cells in a final volume of 200 µL and monitored every 30 min for 72 h by xCELLigence device. The maximum CI corresponds to the minimal lysis (L_min_), tumor cells incubated with irrelevant TCR (OTI-TCR) transduced T cells. The minimum CI corresponds to the maximum lysis, tumor cells co-incubated with 2 mM Staurosporine (Sigma) in the absence of any T cells. Percent lysis, after 12h co-incubation for each sample, was calculated using the following equation, $\%Lysis=\frac{\left(CI_{Lmin}-CI_{Sample}\right)}{CI_{Lmin}}x100$ . Then, the specific lysis for each neoTCR was calculated by normalizing the % Lysis_NeoTCR_ to % Lysis_Staurosporin_ (positive control, 100% lysis).

### 2.4 Statistical analysis and depiction of data

All results are represented with +/- SD of technical duplicates or triplicates. Statistical analysis for each experiment is described in the corresponding figure legend. All statistical analyses were performed using GraphPad PRISM 9 or R version 4.1.0 (47).

## 3 Results

### 3.1 Comparison on genomic level

**Figure 1:**
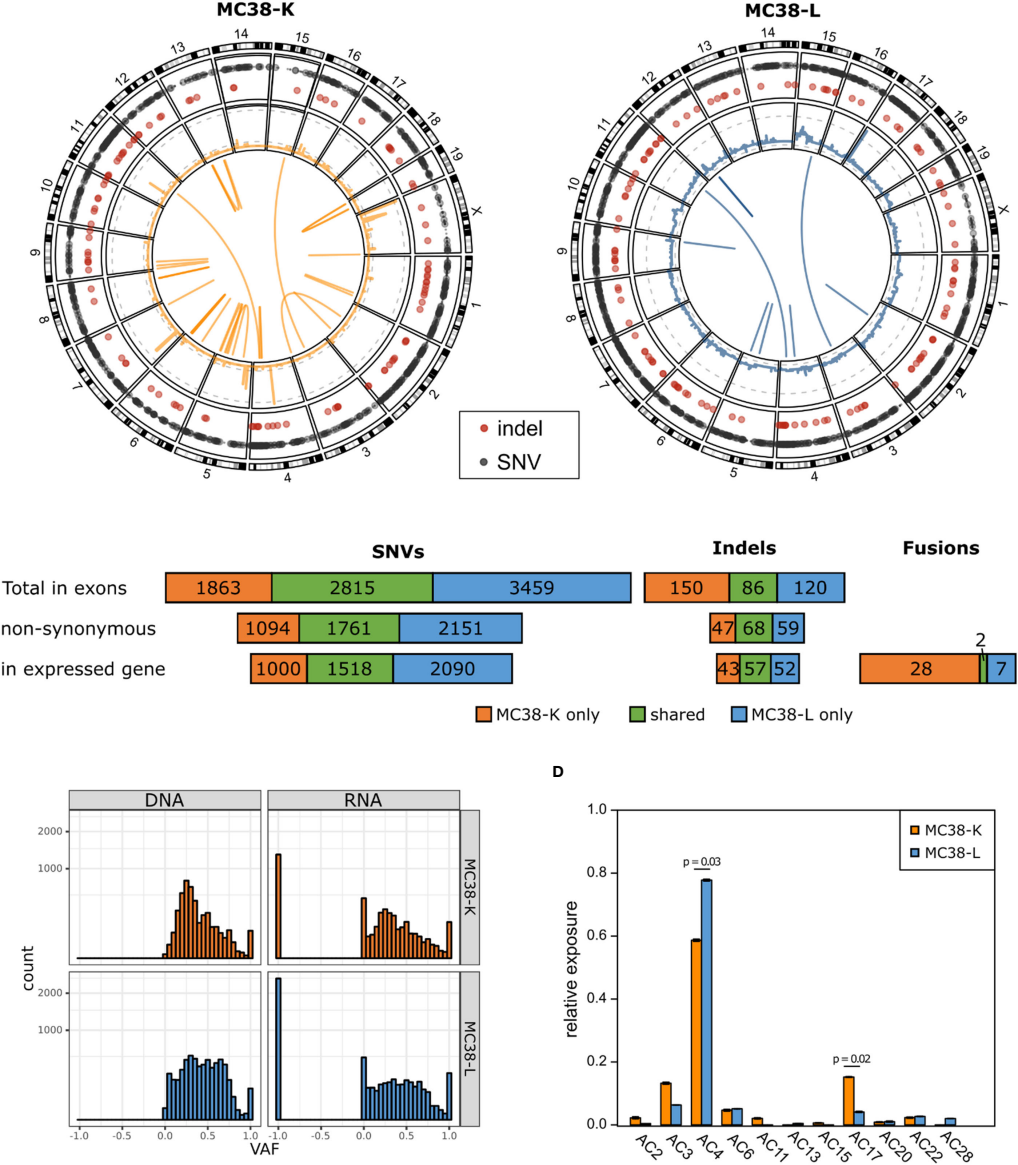
MC38 cell lines MC38-K and MC38-L differ substantially on genomic level. **(A)** Circos plots showing the somatic alterations of both cell lines compared to wild type C57BL/6 mice. Outer circle: SNVs (grey) and small indels (red); second circle from the outside: CNVs, log scaled, with grey dashed lines marking copy numbers 1, 25 and 200 (MC38-K only); middle: fusion gene events. **(B)** Number of SNVs, indels and fusion gene events detected in MC38-K or MC38-L only or shared by both cell lines. **(C)** Variant allele frequencies (VAF) distributions of SNVs in exons in DNA and RNA of both cell lines. VAF values of -1 indicate no coverage in RNA-seq. **(D)** Mutational signatures observed in both cell lines. Significant differences (adjusted p-value < 0.05) are indicated with a line and the respective p-values are depicted. Significance was determined with t-test followed by multiple testing correction with Benjamini-Hochberg correction.

We used whole exome sequencing and RNA-seq data to investigate SNVs and indels (**Supplementary Table S1**), copy number alterations (**Supplementary Table S2**) and fusion genes (**Supplementary Table S3**) in the two MC38 cell lines, MC38-K and MC38-L, and found substantial differences (**Figures 1A, B**). While the MC38-L cell line carried more SNVs and indels, the MC38-K cell line harbored more fusion genes. The overlap was 34.6%, 35.2% and 32.9% for all SNVs in exons, for all non-synonymous SNVs in exons and for all non-synonymous SNVs in exons of expressed genes, respectively (**Figure 1B**). The corresponding values for indels were 24.2%, 39.1% and 37.5%. Only two of in total 37 distinct high confidence fusion gene events (5.4%) were in concordance between the cell lines. Moreover, we observed a distinct structural composition of the genomes under consideration, which is indicated by a high variability of gene copy numbers (**Figure 1A**, middle ring of Circos plot). We determined the ploidy by matching theoretical variant allele frequency (VAF) distributions of SNVs (based on absolute copy numbers, see Methods) with the observed VAF values. This resulted in a ploidy of two for the MC38-K cell line and a ploidy of five for the MC38-L cell line. The number of genes with copy number variants (CNV) included 7,516 and 26,283 genes with a reduced copy number for the MC38-L and MC38-K cell lines, respectively, and 12,864 and 2,659 genes, respectively, with an increased copy number (**Supplementary Table S2**). The resultant absolute gene copy numbers showed no correlation across both cell lines (Pearson correlation coefficient -0.0031). The VAF distributions peaked at 0.25 both in the exome and RNA-seq data of the MC38-K cell line, while the distribution in the MC38-L cell line was more heterogeneous (**Figure 1C**). The observed prevalence of base substitutions was mainly in concordance between the cell lines, but C>T (especially in in TCC and TCT triplets; C is the mutated base, preceded by T and followed by C or T, respectively) and T>G in CTT triplets had a higher relative abundance in the MC38-K cell line compared to other substitutions than in the MC38-L cell line (**Supplementary Figure S1**). In the same line, we observed significant differences in the relative exposure of mutation signatures AC4 (tobacco mutagens, benzoapyrene) which had a higher relative exposure in the MC38-L cell line and AC17 (unknown process) which was stronger in the MC38-K cell line (**Figure 1D**; **Supplementary Table S4**). Signatures AC11 (alkylating agents) and AC15 (defect DNA MMR) was found only in the MC38-K cell line and signatures AC13 (APOBEC) and AC28 (unknown process) were detected only in the MC38-L cell line.

### 3.2 Comparison on transcriptomic level

**Figure 2:**
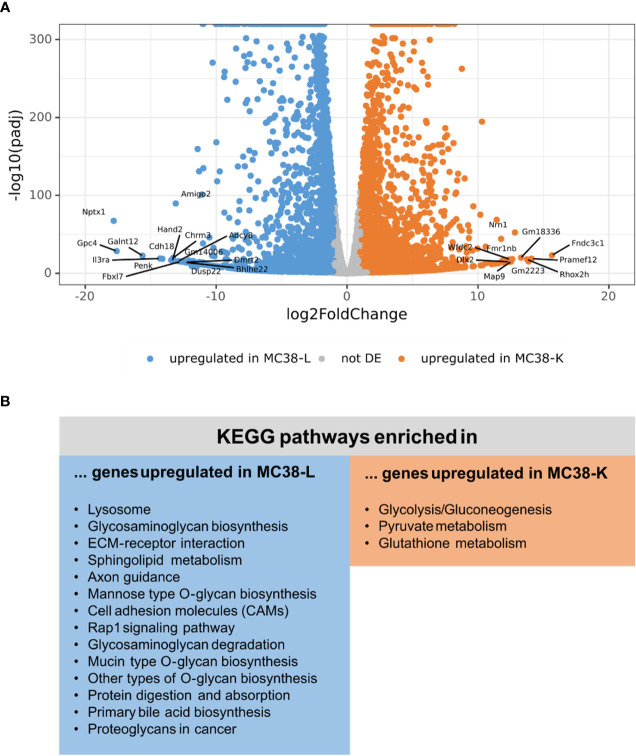
Differential expression analysis of MC38 cell lines indicates distinct transcriptomic profiles. **(A)** Vulcano plot of the differential expression analysis between MC38-K and MC38-L cells. The top 25 differentially expressed (DE) genes are labeled. **(B)** DE genes were subjected to pathway enrichment analysis. Significantly enriched KEGG pathways are shown (adjusted p-value < 0.05).

Next, we compared the expression profiles of the two cell lines. While the normalized count data of the replicates of either cell line had a Pearson’s correlation coefficient of 0.988 (MC38-L) and 0.996 (MC38-K), the correlation coefficient between the cell lines was only 0.952 (**Supplementary Figure S2A**; **Supplementary Table S5**). The mean expression values achieved a correlation coefficient of 0.919 (**Supplementary Figure S2B**) and differential expression analysis between the two cell lines revealed 2,871 genes differentially upregulated in the MC38-K cell line and 9,252 genes differentially upregulated in the MC38-L cell line (absolute log2foldchange > 1, adjusted p-value < 0.05; **Figure 2A**; **Supplementary Table S6**). The genes that were upregulated in the MC38-L cell line were significantly enriched for genes involved in various KEGG (Kyoto Encyclopedia of Genes and Genomes) pathways including lysosome, glycosaminoglycan biosynthesis, ECM-receptor interaction, sphingolipid metabolism, axon guidance, mannose type O-glycan biosynthesis, and cell adhesion molecules (CAMs) (adjusted p-value < 0.05, **Figure 2B**). The enriched pathways were associated with different biosynthesis processes and processes regulating cell adhesion, cell-cell junction formation and cell polarity. The KEGG pathways that were significantly enriched in the genes upregulated in the MC38-K cell line were glycolysis/gluconeogenesis, pyruvate metabolism, and glutathione metabolism (**Figure 2B**).

### 3.3 Comparison on immunogenic level

**Figure 3:**
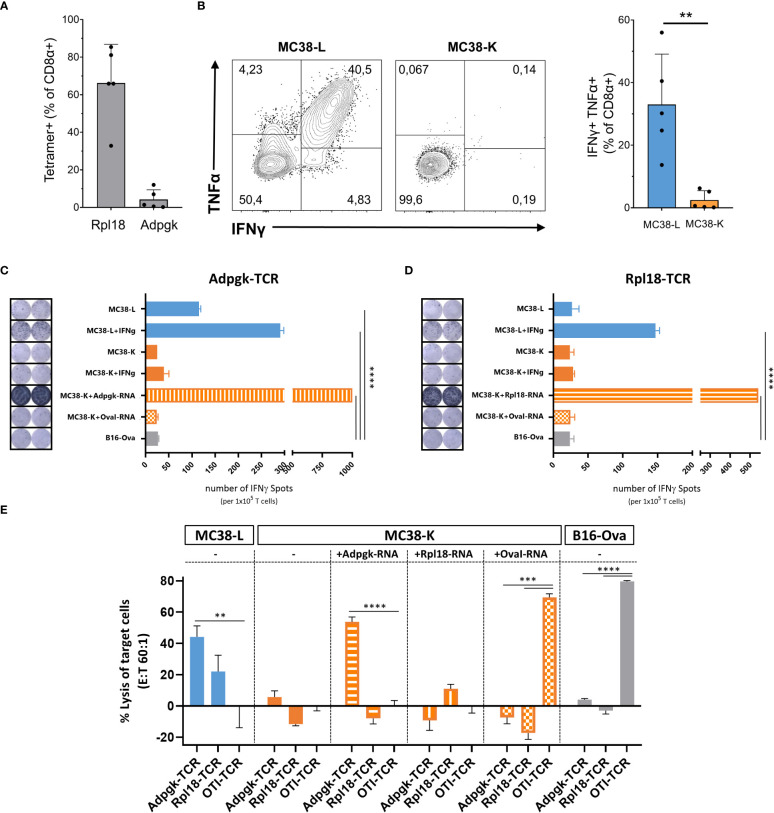
Comparison of MC38-L and MC38-Kcell lines on immunogenic level. **(A)** Antigen specificities of established CD8+ T cell lines were analyzed by Rpl18 and Adpgk specific tetramers. **(B)** Established CD8+ T cell lines were analyzed for recognition of MC38-L or MC38-K tumor cells by induced cytokine production after coculture with live tumor cells. IFNγ secretion by Adpgk-TCR **(C)** or Rpl18-TCR **(D)** transduced T cells upon co-culture with different tumor cells *via* ELISPOT assay. Data indicate mean ± SD of biological replicates (n=2). P values determined by One-way ANOVA Tukey´s multiple comparison test. **(E)** *In vitro* cytotoxic activity of Adpgk-TCR or Rpl18-TCR transduced T cells after 12h co-culture with different tumor cells. Data indicate mean ± SD of biological replicates (n=3). P-values were determined with respect to OTI-TCR control by One-way ANOVA Bonferroni´s multiple comparison test. **p < 0.01, ***p < 0.001, ****p < 0.0001.

Despite both MC38-L and MC38-K cell lines being of the same origin, MC38, and possessing some mutations in common, they can be distinguished based on the expression of cell-line specific mutations such as Adpgk_R304M_ and Rpl18_Q125R_ (**Table 1**). The mutations in Adpgk and Rpl18 induced endogenous CD8+ T cell responses when MC38-L tumors regressed in mice treated with αPDL1 and splenocytes were expanded *ex vivo* upon recurrent stimulation with irradiated MC38-L cells (**Figure 3A**) to generate antigen-specific CD8+ T cell lines. Coculture of established Adpgk_R304M_ or Rpl18_Q125R_ specific CD8+ T cell lines (**Figure 3A**) with MC38-L and MC38-K cells showed a strongly reduced capacity of the T cells to recognize MC38-K cells (**Figure 3B**).

**Table 1:** Expression of previously published (candidate) neoantigens in the MC38-K and MC38-L cell lines.

Gene symbol	Mutation	Amino acid exchange	Mutated sequence	Transcript expression		Variant allele frequency		Variant expression	
				K	L	K	L	K	L
Adpgk*	chr9:59161630	R304M	HLELASMTN**M**ELMSSIVHQ	7.24	30.61	–	0.34	–	10.43
Gtf2i	chr5:134739515	G396V	FRRPSTY**V**IPRLERILLAK	27.27	14.54	–	0.48	–	6.93
Reps1*	chr10:17775901	P45A	RVLELFRAAQL**A**NDVVLQIME	23.54	19.83	–	0.18	–	3.52
Rpl18 *	chr7:52975740	Q125R	KAGGKILTFD**R**LALESPK	1139.01	1103.60	–	0.38	–	414.82
Wbp11	chr6:136770171	V134L	QYFDAVKNAQH**L**EVESIPLPD	51.55	35.66	–	0.24	–	8.59
Aatf	chr11:84256087	A500T	SFMAPIDHT**T**MSDDARTE	39.06	28.84	0.21	0.46	8.12	13.24
Cpne1	chr2:155903380	D302Y	GSNGDPSSP**Y**SLHYLSPTGVNE	66.44	20.69	0.15	0.38	10.01	7.77
Dpagt1*	chr9:44137208	V213L	EAGQSLVISASIIVFNL**L**ELEGDYR	22.11	27.68	0.32	0.55	7.12	15.12
Tdg	chr10:82110022	Q269L	ARCAQFPRA**L**DKVHYYIKLKD	33.03	21.67	0.28	0.60	9.10	13.11

The listed neoantigens were reported by Hos et al. (20) and Yadav et al. (14). The mutated amino acid in the mutated sequence is indicated in bold. The variant expression calculated as variant expression = transcript expression x variant allele frequency. (K: MC38-K, L: MC38-L). *: CD8^+^ T cell activation observed by Hos et al. (20) and/or Yadav et al. (14). -: mutation not detected.

To further explore this difference between MC38-L and MC38-K cells on the immunological level, we engineered T cells expressing TCRs against Adpgk_R304M_ or Rpl18_Q125R_ neoantigens and evaluated IFNγ secretion as well as cytotoxicity by TCR-specific T cells upon co-culture with tumor cells. Upon stimulation, Adpgk-TCR transduced T cells recognized MC38-L but not MC38-K cells (**Figure 3C**). After co-culture with IFNγ pre-stimulated MC38-L cells, Rpl18-TCR transduced T cells also showed tumor recognition (**Figure 3D**). IFNγ pre-stimulation of MC38-L cells prior to co-culture with TCR-transduced T cells resulted in an increase (>50%) in the number of IFNγ spots (**Figures 3C, D**). The number of IFNγ spots was comparable between MC38-K, with or without IFNγ pre-stimulation, and B16-Ova cells, our control cell line, pointing out that MC38-K cell line is not recognized by T cells of Adpgk_R304M_ or Rpl18_Q125R_ neoantigens specificity. Only forced expression of these neoantigens but not OvaI_257-264_ in MC38-K cells *via* electroporation of matching neoantigen encoding RNAs resulted in significant recognition of the tumor cells by Adpgk- or Rpl18-TCR transduced T cells (**Figures 3C, D**).

Following tumor cell recognition *via* IFNγ ELISPOT, we also tested *in vitro* cytotoxic effects of TCR-transduced T cells on tumor cells. Adpgk- and Rpl18-TCR transduced T cells resulted in 40% and 20% lysis of MC38-L cells, respectively (**Figure 3E**). TCR-transduced T cells caused lysis of MC38-K cells only when these cells were forced to express the matching antigens for the TCRs. Otherwise, the percentage of lysed cells by neoantigen-specific TCRs was similar between MC38-K and B16-Ova cells.

## 4 Discussion

Murine tumor cell lines are a well-established tool for preclinical studies. MC38 is among the most commonly used tumor models for colorectal carcinoma and can be regarded as a “workhorse” for cancer immunotherapy research. Accordingly, MC38 is currently mentioned in more than 500 articles listed in Pubmed (search term “((mc-38) OR mc38) AND tumor AND model”, 31MAY2022). By analyzing MC38 cells from two different sources, we revealed that there are at least two sub-cell lines. The two cell lines have a distinct genomic composition, distinct mutational signatures and share a minor portion of their non-synonymous variants (SNVs, indels) and fusions (35.3% and 5.4% respectively). This is in a similar range to that reported in a previous study in a series of human MCF7 breast cancer cell lines (48).

The expression profiles of MC38-K and MC38-L cells correlated strongly, but there were still notable differences. Cell culture conditions can influence expression profiles, but the effect that we observed was very prominent with several thousands of genes being differentially upregulated in either cell line (MC38-K: 2,871 genes; MC38-L: 9,252 genes). Using a reduced representation of the transcriptome that allows to infer 81% of non-measured transcripts [“L1000 assay” (49)], Ben-David and colleagues (48) found a median of 654 genes (range: 10–1,574) that were differentially expressed by at least two-fold in pairs of MCF7 cell lines. Of note, Adpgk and Rpl18 were not differentially expressed in our analysis (**Supplementary Table S6**). Thus, both neoantigens would have the same potential to be recognized by T cells but the mutations were only present in MC38-L cells. Furthermore, we found the endogenous retroviral element gp70 to be highly expressed in both cell lines (696.6 RPKM and 1977.7 RPKM in MC38-K and MC38-L, respectively). Since also the RNA-seq data confirmed homogeneous coverage across the whole transcript, one can expect that the dominant epitope KSPWFTTL (50) as well as any other potential epitopes expressed from this transcript will be present in both cell lines.

With the transfection of the neoepitope-specific TCRs in T cells, we confirmed our findings that T cell lines raised on MC38-L cells induce expansion of Adpgk_R304M_ and Rpl18_Q125R_ specific T cells with specificity for MC38-L tumor cells while non-responsive to MC38-K cells. Induced expression of the mutated peptides by transfection rescues the recognition of MC38-K cells by the transduced T cells, thus reaffirming the lack of the mutations as the key reason for the absence of recognition of the MC38-K cells.

We further screened literature for exemplary studies addressing immunotherapeutic strategies in MC38. Yadav et al. (14) trace back their cells to “Academisch Ziekenhuis Leiden” (or Academic Hospital Leiden, now named: Leiden University Medical Center) and the observed mutational burden is in concordance with what we found for the MC38-L cells. Zhong and colleagues (51) refer to the laboratory of Antoni Ribas at UCLA, LA, California. The sequenced *ex vivo* tumor material shows a mutational profile (base substitutions, mutational load) similar to our MC38-L cells. Furthermore, they find Smad4 mutated which we detected also only in MC38-L cells. Other studies [e.g (52).] name Kerafast as the source of their MC38 cells, but use Yadav et al. (14) as the reference for neoantigens for their peptide vaccination. In that manuscript, the neoantigen Dpagt1^mut^ which is present in MC38-K and MC38-L cells was included in the peptide pool for vaccination. Hence, immune responses could still be observed.

Given the genetic instability and variability of tumor cell lines in general, our analyses further underline the importance of accurate tracing of tumor cell lines in the experimental design to ensure reproducible studies and avoiding artifact in data interpretation due to genomic (and thus transcriptomic as well as immunogenic) differences.

## Data availability statement

The datasets presented in this study can be found in online repositories. The names of the repository/repositories and accession number(s) can be found below: https://www.ebi.ac.uk/ena, PRJEB56522.

## Ethics statement

Ethical review and approval was not required for the animal study because the research proposal was approved by the local ethics committee of the Government of Rhineland Palatinate, Germany.

## Author contributions

US, FO and MD conceived and guided the study. BH, MV and IY performed and analyzed experiments. JB was responsible for sequencing. BS, ML, FL and CH performed bioinformatics analyses. BS, ML, BH, MV, FO and MD interpreted the results. BS, BH, FO and MD wrote the manuscript. All authors contributed to the article and approved the submitted version.
