# Supplementary material for: MC38 colorectal tumor cell lines from two different sources display substantial differences in transcriptome, mutanome and neoantigen expression
Source: Front Immunol. 2023 Mar 8;14:1102282. doi: 10.3389/fimmu.2023.1102282 (PMC10030996; doi:10.3389/fimmu.2023.1102282)
Supplement: Supplementary file 1 [file DataSheet_1.docx]

Supplementary Material

## Supplementary Figures


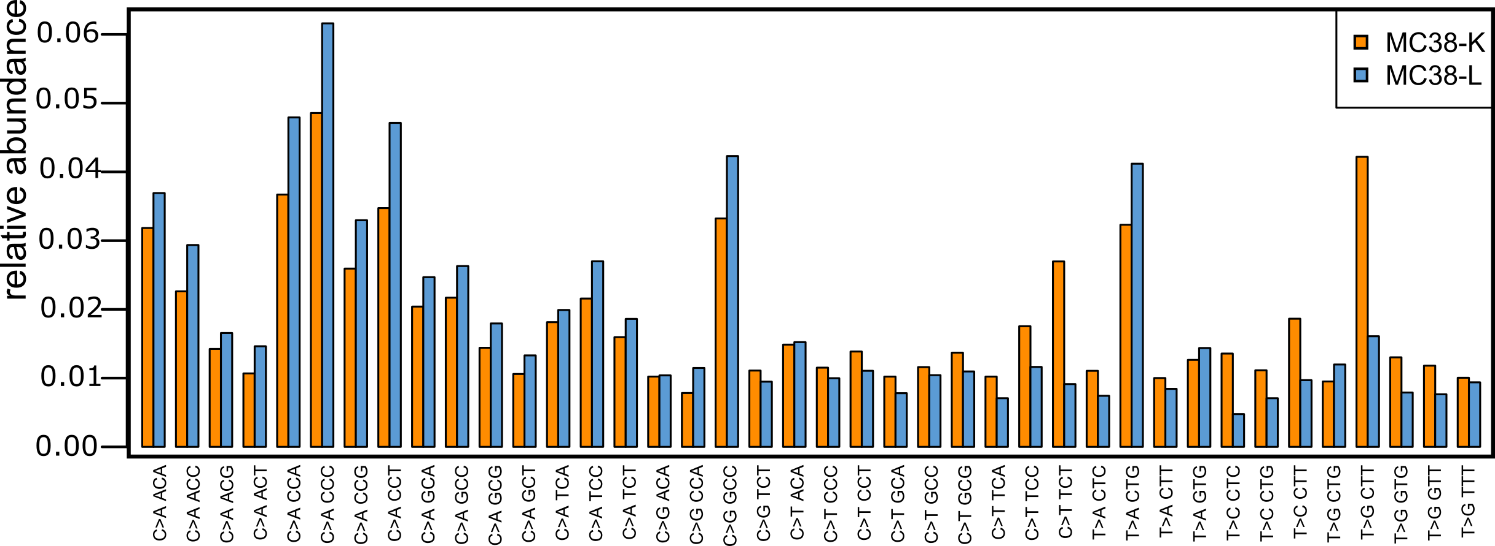


**Supplementary Figure S1:** Abundance of nucleotide substitutions in both cell lines with respect to nucleotide triplets. Only combinations with a relative abundance > 0.01 in at least one of the cell lines are shown.


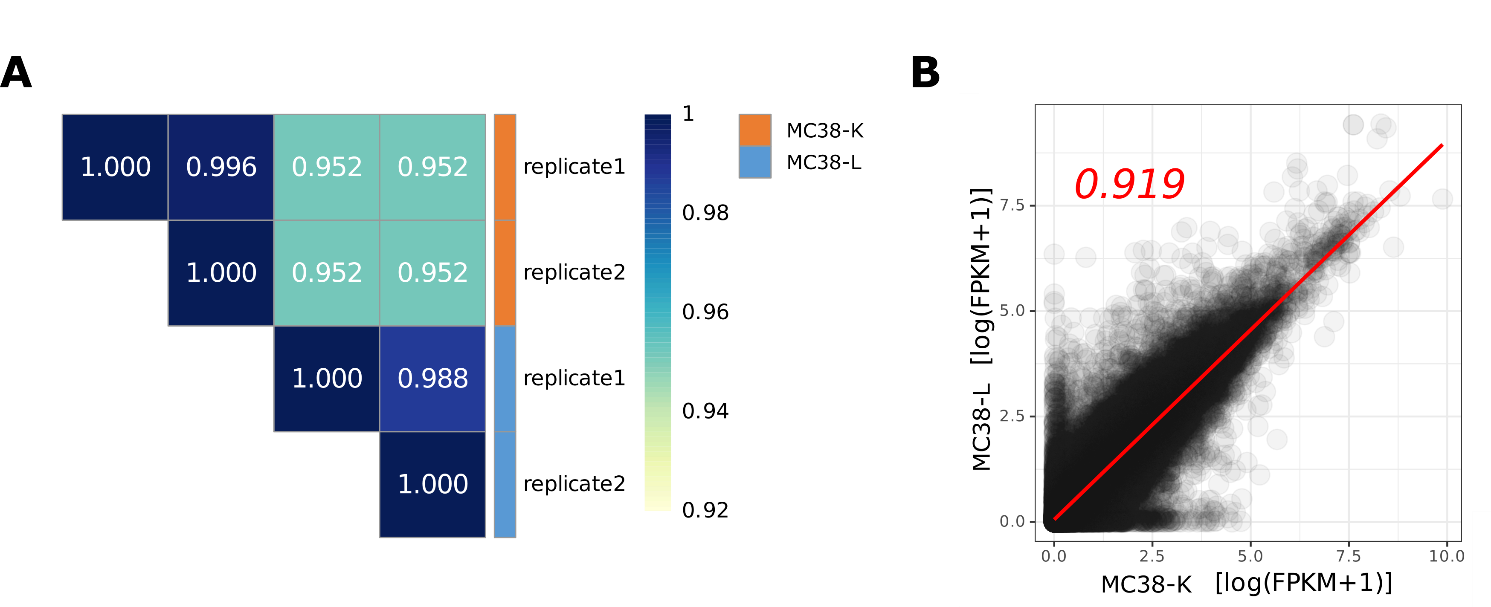


**Supplementary Figure S2: Correlation of expression profiles. (A)** Pearson’s correlation coefficients of normalized count data between sequencing replicates and between MC38-K and MC38-L cell lines. **(B)** Scatter plot of log-transformed FPKM expression values (mean of replicates) of MC38-K and Leiden cells. The red line MC38-L the linear regression (correlation coefficient: 0.919).

## Supplementary Tables

**Supplementary Table S1: SNVs and small insertions and deletions (indels) detected in MC38-K (A) and MC38-L (B).**

**Supplementary Table S2: Absolute copy numbers (CN) determined in MC38-L and MC38-K.**

**Supplementary Table S3: High confidence fusion genes detected MC38-K and MC38-L.** Breakpoints and breakpoint IDs (BPID) are indicated in GRCm38.95 coordinates. One BPID can be associated with more than one Fusion_Gene. The columns "ft_junc_cnt_best_per_FTID" and "ft_span_cnt_best_per_FTID" refer to the number of junction and spanning reads supporting each fusion transcript listed in the column "FTIDs" separated by "|". The column "prediction_prob_per_FTID" likewise indicates the prediction scores provided by EasyFuse.

**Supplementary Table S4: Comparison of relative abundance of mutational signatures in both MC38 cell lines.** Significant differences between the cell lines were determined with t-test followed by multiple testing correction with Benjamini-Hochberg correction.

**Supplementary Table S5: Mean transcript expression values in RPKM of MC38-K and MC38-L.**

**Supplementary Table S6: Differential expression analysis between MC38-L and MC38-K.** Shrunken log2FoldChange (type "apeglm") are listed. A positive log2FoldChange indicates upregulation in the MC36-K cell line, a negative log2Fold Change indicates downregulation in MC38-K cell line.
